# The effects of high-intensity interval training on glucose metabolism, inflammatory responses, and functional recovery in patients with diabetic peripheral neuropathy

**DOI:** 10.3389/fendo.2026.1879941

**Published:** 2026-06-18

**Authors:** Genchun Guo, Zhenhua Zhu, Xin Shao, Feixiang Ma, Chunyan Xing, Yunlan Huang, Wanlang Li

**Affiliations:** Department of Rehabilitation Medicine, Affiliated Hospital 6 of Nantong University (Yancheng Third People’s Hospital, the Affiliated Hospital of Jiangsu Medical College), Yancheng, China

**Keywords:** diabetes, glucose metabolism, high-intensity interval training, inflammatory responses, moderate-intensity continuous training, peripheral neuropathy

## Abstract

**Objective:**

The aim of this study is to investigate, through a randomised controlled trial, the effects of high-intensity interval training (HIIT) on glucose metabolism, inflammatory responses and neurological recovery in patients with diabetic peripheral neuropathy (DPN), and to compare these effects with those of moderate-intensity continuous training (MICT), thereby providing new and more effective evidence-based guidance for the exercise rehabilitation of DPN.

**Methods:**

A total of 93 patients with DPN were randomly assigned to the HIIT, MICT, and control (CON) groups, with an intervention period of 12 weeks. Fasting plasma glucose (FPG), glycated hemoglobin (HbA1c), insulin resistance index (HOMA-IR), tumor necrosis factor-α (TNF-α), interleukin-6 (IL-6), C-reactive protein (CRP), motor nerve conduction velocity (MNCV), sensory nerve conduction velocity (SNCV) of the common peroneal nerve, Toronto Clinical Neuropathy Score (TCSS), and Berg Balance Scale.

**Results:**

Compared with CON, both HIIT and MICT improved HbA1c, FPG, and HOMA-IR levels to some extent, reduced the expression of inflammatory factors such as TNF-α, IL-6, and CRP, and promoted the recovery of nerve conduction velocities and clinical function. Compared with MICT, HIIT demonstrated more significant and sustained advantages in terms of HbA1c, FPG, TNF-α, IL-6, and TCSS scores.

**Conclusion:**

HIIT and MICT can effectively improve glucose metabolism, reduce chronic inflammatory responses, and promote the recovery of nerve function in patients with DPN; however, HIIT is superior to MICT in terms of the extent of improvement, sustainability, and overall benefits.

## 1 Introduction

Diabetic peripheral neuropathy (DPN) is the most common chronic complication of diabetes, with a higher prevalence than diabetic nephropathy and retinopathy (1). The progression of DPN is insidious; it initially affects the sensory function of the peripheral nerves, with typical symptoms including hyperesthesia, loss of sensation, or abnormal sensations in the toes and feet on both sides. Subsequently, the condition gradually extended upward to the legs, presenting a symmetrical sock-and-stocking distribution. Motor symptoms develop secondary to sensory disturbances; in the early stages, these manifest as diminished or absent ankle tendon reflexes, and as neuropathy progresses, weakness of the toe muscles and abnormal knee reflexes may occur (2, 3). The pathogenesis of DPN is closely associated with metabolic disturbances, oxidative stress, and low-grade inflammation resulting from long-term chronic hyperglycemia. Persistent low-grade inflammation can damage vascular endothelial cells, reduce neural blood supply, and directly or indirectly lead to demyelination of nerve fibers and axonal degeneration (4, 5). Patients with DPN are significantly affected in terms of daily living and occupational functioning. In addition to physical symptoms, they often experience reduced mobility, an increased risk of falls, sleep disturbances, and psychological distress, such as anxiety and depression, with a continuous decline in their overall health status and quality of life. Multiple studies have demonstrated that DPN is the key pathological basis for complications such as diabetic foot ulcers and Charcot neuroarthropathy, which increase the risk of lower limb amputation. Even after adjusting for potential confounding factors, DPN remains an independent predictor of poor long-term prognosis (6–8). Relevant studies indicate that the mortality rate among patients with foot ulcers is approximately 23% within two years, rising to 71% within ten years, with extremely low long-term survival rates (9).

DPN is not only a metabolic complication but also a neuromuscular disorder characterized by impaired motor unit recruitment, reduced muscle strength, altered neuromuscular coordination, loss of proprioception, and impaired postural control. These neuromuscular dysfunctions are largely responsible for gait instability, reduced mobility, and increased risk of falls in patients with DPN. Recent research further indicates that exercise intervention helps to stabilize blood glucose control, promote neural blood perfusion, alleviate oxidative stress and inflammatory responses, optimize neural metabolic status, and improve the structure and function of nerve fibers, thereby delaying the onset and progression of DPN (10). Furthermore, it can enhance overall functional capacity and postural control, improve balance and gait stability, and consequently improve patients’ ability to perform activities of daily living and reduce the risk of falls (11). Consequently, exercise-based rehabilitation strategies may offer multidimensional benefits to patients with DPN through dual metabolic and neuromuscular mechanisms. According to the relevant guidelines of the American Diabetes Association, patients with type 2 diabetes should engage in at least 150 min of moderate-intensity or 75 min of vigorous-intensity physical activity per week (12). Building on this, previous studies have further demonstrated that, compared with moderate-intensity continuous training (MICT), high-intensity interval training (HIIT) can induce comparable or even more significant cardiovascular adaptations and glycemic metabolic benefits, improving aerobic fitness, body fat content and distribution, reducing blood glucose and lipid levels, enhancing insulin sensitivity, and improving microvascular perfusion and neural blood supply (13, 14). However, given the risk of sensory impairment and foot ulcers in patients with diabetic peripheral neuropathy (DPN), HIIT has been cautiously applied in this population, and its safety, efficacy, and specific effects on neurofunction remain unclear. Therefore, this study aimed to investigate the effects of HIIT on glucose metabolism, inflammatory responses, and neurofunctional recovery in patients with DPN through a randomized controlled trial, comparing it with MICT to provide new and more effective evidence-based guidance for exercise rehabilitation in DPN.

## 2 Materials and methods

### 2.1 Sample size estimation

The sample size was estimated using G*Power 3.1 software, based on previous literature (15) and using the change in FPG as the primary outcome measure. An F-test was set up, and the following ANOVA was selected: fixed effects, omnibus, and one-way. Using *a priori* analysis, the effect size was set at 0.4, α at 0.05, and power at 0.90, with three groups and two degrees of freedom in the numerator. The target sample size was determined to be 84. Considering a 10% natural attrition rate, this study plans to enrol 93 eligible participants.

### 2.2 Study participants

A total of 93 patients with type 2 diabetes and DPN who were treated at Yancheng Third People’s Hospital between January 2024 and July 2025 were consecutively enrolled. Eligible participants were randomized using a random number table into the HIIT, MICT, and CON, comprising 31 participants in each group. This study was approved by the Medical Ethics Committee of Yancheng Third People’s Hospital (Ethics Approval No. 2024-93) and registered with the Chinese Clinical Trial Registry (ChiCTR2500109646). All the enrolled participants provided written informed consent.

The inclusion criteria were as follows: (1) a diagnosis of diabetic peripheral neuropathy (DPN) according to the Expert Consensus on the Diagnosis and Treatment of Diabetic Neuropathy (2021 edition), based on the presence of diabetes mellitus together with neuropathic symptoms and/or signs after exclusion of other causes of peripheral neuropathy (16); (2) Toronto Clinical Scoring System (TCSS) score ≥6; (3) regular moderate-intensity physical activity duration <30 min per week; (4) stable condition with no major adjustments to the glycemic control regimen within the past 3 months. Nerve conduction studies were performed at baseline for all participants to characterize their neurological status; however, electrophysiological abnormalities were not required for study enrollment and were evaluated as outcome measures during the trial.

The exclusion criteria were as follows: (1) severe cardiovascular or cerebrovascular disease (e.g., unstable angina, myocardial infarction within the past 6 months, heart failure, and NYHA class III–IV) or uncontrolled hypertension (resting blood pressure ≥160/100 mmHg); (2) severe diabetic complications (e.g., proliferative retinopathy, diabetic foot ulcers, clinical proteinuria, or renal insufficiency); (3) peripheral neuropathy caused by other factors (e.g., cervical spondylosis, lumbar disc herniation, alcoholism, and vitamin B12 deficiency); (4) musculoskeletal disorders or contraindications preventing the completion of exercise testing; and (5) cognitive impairment or psychiatric disorders preventing participation in the study.

Discontinuation and withdrawal criteria were as follows: (1) occurrence of serious adverse events or exercise-related injuries during the study; (2) poor compliance or withdrawal requested by the participant for personal reasons; and (3) significant changes to the participant’s blood glucose management regimen during the intervention period that may affect the interpretation of the study results.

### 2.3 Study design

This study established a multidisciplinary exercise management team comprising rehabilitation physicians, cardiologists, rehabilitation therapists, and nursing staff. The team’s primary responsibilities included developing, reviewing, and dynamically optimizing the exercise intervention program; monitoring key parameters, such as blood glucose and blood pressure, during the intervention; distributing and collecting study materials; providing personalized exercise guidance and training on the correct use of exercise equipment; assessing and adjusting exercise intensity; educating participants on exercise-related precautions; administering and managing follow-up questionnaires; and organizing, entering, and quality-controlling research data. Should any issues arise during the study, the team will promptly consult specialist physicians and handle them in accordance with established protocols to ensure the safety of the intervention. All training sessions were conducted on the Monark 834E magnetic power bike, a device capable of precisely setting and displaying power (watts, W), thereby ensuring the objectivity and reproducibility of exercise intensity. Prior to the intervention, all participants underwent a graded exercise test to determine their individual peak power output (PPO) and maximum heart rate (HRmax) values. All exercise intensities were set based on the individualized data. Fingerstick blood glucose was monitored before each exercise session, during exercise (every 10 minutes), and immediately after exercise. If blood glucose levels were < 5.6 mmol/L or > 16.7 mmol/L, the day’s training was suspended. Sweets and glucose tablets were available prior to exercise; should hypoglycemic symptoms such as palpitations, cold sweats, or dizziness occur during exercise, the session was immediately halted and appropriate measures were taken. Each training session for both groups included a 5-minute warm-up (low-intensity cycling and dynamic stretching) and a 5-minute cool-down (low-intensity cycling and static stretching) to prevent exercise-related injuries and promote recovery. Supervisors used Polar H10 heart rate monitors to continuously monitor and record the participants’ real-time heart rates. After each training session, the participants’ blood glucose levels before, during, and after exercise were recorded, along with the average heart rate, maximum heart rate, subjective fatigue levels (Rating of Perceived Exertion, RPE, using a 6–20 point Borg scale), and any symptoms of discomfort or adverse events.

HIIT Group: A 1:1 interval training protocol was adopted and conducted three times a week, with at least one day’s rest between sessions. The total duration was approximately 40 min, beginning with a 5-minute warm-up (50% PPO), followed by the main exercise phase. This phase comprised three sets of training units, each consisting of four cycles; each cycle involved 1 min of high-intensity exercise (at 90% PPO, with a target heart rate maintained at 85–90% HRmax) and 1 min of active recovery (load reduced to 50% PPO, target heart rate reduced to 60–70% HRmax). A 2-minute complete rest period (ceasing pedalling) followed each set, with the session concluding with a 5-minute cooldown (50% PPO). The entire program involved reassessment and progressive adjustment of exercise load every 2 weeks based on the patient’s tolerance and subjective fatigue (RPE score) (17).

MICT Group: This group followed a moderate-intensity continuous training regimen, with sessions three times a week, spaced at least one day apart. The total duration was approximately 40 min, beginning with a 5-minute warm-up (50% PPO), followed by 30 min of constant-load moderate-intensity exercise (load set at 50–60% PPO, with target heart rate maintained at 60–70% HRmax), and concluding with a 5-minute cool-down (50% PPO). As with the HIIT group, this program involved reassessing and fine-tuning the exercise load every two weeks based on patient tolerance and subjective fatigue to ensure that the intensity was consistently maintained within the target range.

The control group maintained their usual daily activities, received health education, and underwent telephone follow-ups every four weeks but did not participate in a structured exercise program.

### 2.4 Collection of primary data

The following data were collected by the same trained assessor, who was unaware of the group allocation, at baseline (prior to the intervention), at the end of the intervention (week 12), and at the end of follow-up (week 24).

#### 2.4.1 Primary outcomes

Glucose metabolism parameters: Fasting plasma glucose (FPG) was measured using a fully automated biochemical analyzer; glycated hemoglobin (HbA1c) and the Homeostatic Model Assessment of Insulin Resistance (HOMA-IR) were measured using high-performance liquid chromatography (HPLC); HOMA-IR = FPG × fasting insulin (FINS) ÷ 22.5, with higher values indicating a greater degree of insulin resistance.

#### 2.4.2 Secondary outcomes

(1) Inflammatory markers: Five milliliters of venous blood was collected in the morning on an empty stomach, centrifuged at 3000 rpm for 10 min, and the supernatant was transferred to a -80 °C freezer for storage pending analysis. Serum concentrations of tumor necrosis factor-α (TNF-α) and interleukin-6 (IL-6) were measured using enzyme-linked immunosorbent assay (ELISA), and high-sensitivity C-reactive protein (hs-CRP) concentrations were measured using immunoturbidimetric assay.

(2) Neurological assessment: Using the NeuroCare-D1 electromyography and evoked potential system, the motor nerve conduction velocity (MNCV) and sensory nerve conduction velocity (SNCV) of the common peroneal nerve were measured while maintaining skin temperature at 32 °C or above.

(3) Clinical symptoms and function: The Toronto Clinical Neuropathy Scale (TCSS) and Berg Balance Scale were used.

### 2.5 Statistical analysis

Statistical analyses were performed using SPSS version 26.0 (IBM Corp., Armonk, NY, USA) and GraphPad Prism version 10.2.1 (GraphPad Software, San Diego, CA, USA). Normality of continuous variables was assessed using the Shapiro–Wilk test, and homogeneity of variance was evaluated using Levene’s test. Normally distributed continuous variables are presented as mean ± standard deviation (SD), and baseline between-group comparisons were performed using one-way analysis of variance (ANOVA). Non-normally distributed variables are expressed as median and interquartile range [median (IQR25, IQR75)] and were analyzed using the Kruskal–Wallis test. Categorical variables are presented as frequencies and percentages [n (%)] and were compared using the chi-square test or Fisher’s exact test, as appropriate. Repeated-measures ANOVA was used to evaluate changes over time and differences among groups for primary outcome measures, including HbA1c, FPG, HOMA-IR, TNF-α, IL-6, CRP, MNCV, SNCV, TCSS, and Berg score. Time effects, group effects, and time × group interactions were assessed. Mauchly’s test was used to examine sphericity, and Greenhouse–Geisser correction was applied when necessary. Bonferroni correction was used for *post hoc* multiple comparisons. P value < 0.05 was considered statistically significant.

## 3 Results

### 3.1 Participant characteristics

A total of 105 potential participants were screened for this study, of whom 93 DPN patients meeting the inclusion criteria were initially randomised, with 31 participants in each of the HIIT group, the MICT group and the control group. During the 12-week intervention period and the subsequent 12-week follow-up period, seven participants withdrew from the study(HIIT: n=2; MICT: n=2; CON: n=3) due to personal reasons, scheduling conflicts or an inability to complete the follow-up assessments. Consequently, 86 participants completed the study and had complete outcome datasets available for final analysis. There were no statistically significant differences between the three groups in terms of age, sex, duration of diabetes, body mass index, or any of the baseline outcome measures (P > 0.05), indicating that the groups were comparable (**Table 1**).

**Table 1:** Comparison of baseline characteristics among the three patient groups.

Variables		HIIT (n=29)	MICT (n=29)	CON (n=28)	*t/χ²*	*P*
Age (years)		64.95 ± 6.78	65.33 ± 6.82	65.16 ± 7.01	0.47	0.676
Gender, n (%)	Male	13 (44.8)	12 (41.4)	12 (42.9)	0.73	0.782
	Female	16 (55.2)	17 (58.6)	16 (57.1)		
Body Mass Index (kg/m2)		24.35 ± 2.31	23.88 ± 2.46	24.13 ± 2.51	0.25	0.345
Duration of illness (years)		6.36 ± 2.85	6.61 ± 2.37	6.53 ± 2.96	0.76	0.833

### 3.2 Primary outcome measures

Comparison of glucose metabolism parameters at different time points.

**Figure 1:**
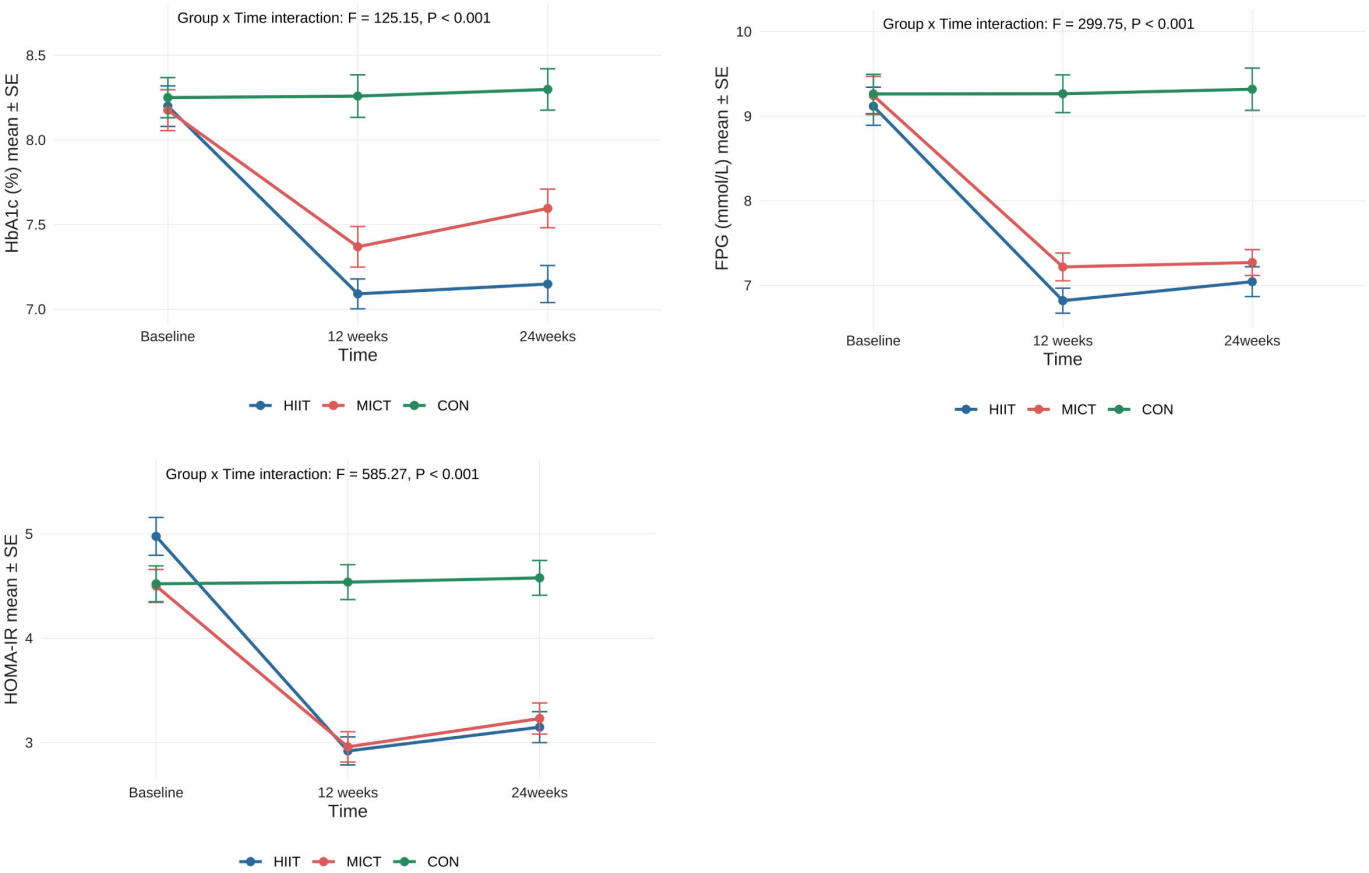
Trend chart showing the mean glucose metabolism at various time points.

The results of repeated measures analysis of variance indicated significant interaction effects between the time factor and intervention group for HbA1c, FPG, and HOMA-IR (all P < 0.001) (**Table 2** and **Figure 1**), suggesting that the effects of the various interventions on glucose metabolism parameters exhibited different trends over time. Further analysis using Bonferroni *post-hoc* tests revealed that, compared with pre-intervention levels, the HIIT and MICT groups exhibited significant reductions in HbA1c, FPG, and HOMA-IR at the 12-week and 24-week follow-up visits (all P < 0.05), whereas no significant changes in these parameters were observed in the control group at any time point (all P > 0.05). Analysis of between-group effects showed that, compared with the control group, the HIIT and MICT groups exhibited significant reductions in HbA1c, FPG, and HOMA-IR at the 12-week and 24-week follow-ups (all P < 0.05). Comparedwith the MICT group, both HbA1c and FPG in the HIIT group decreased significantly at the 12-week intervention (all P < 0.05), and HbA1c in the HIIT group at the 24-week follow-up was lower than that in the MICT group (P < 0.05).

**Table 2:** Comparison of glucose metabolism outcomes among HIIT, MICT, and CON.

Variables	Baseline	12 weeks	24 weeks	Time×group F (P)	Partial η²	95% CI
HbA1c (%)				125.153 (<0.001)	0.751	0.695-1.000
HIIT	8.20 ± 0.64	7.09 ± 0.48^abc^	7.15 ± 0.59^abc^			
MICT	8.18 ± 0.65	7.37 ± 0.65^ab^	7.60 ± 0.61^ab^			
CON	8.25 ± 0.63	8.26 ± 0.66	8.30 ± 0.65			
FPG (mmol/L)				299.745 (<0.001)	0.878	0.847-1.000
HIIT	9.12 ± 1.21	6.82 ± 0.80^abc^	7.04 ± 0.95^ab^			
MICT	9.24 ± 1.21	7.22 ± 0.88^ab^	7.27 ± 0.82^ab^			
CON	9.26 ± 1.23	9.27 ± 1.18	9.32 ± 1.32			
HOMA-IR				585.271 (<0.001)	0.934	0.918-1.000
HIIT	4.98 ± 0.98	2.92 ± 0.72^ab^	3.15 ± 0.80^ab^			
MICT	4.50 ± 0.85	2.96 ± 0.78^ab^	3.23 ± 0.81^ab^			
CON	4.52 ± 0.91	4.54 ± 0.88	4.58 ± 0.88			

Compared with pre-intervention levels within the group,

^a^ P < 0.05; compared with the control group at the same time point,

^b^ P < 0.05; compared with the MICT group at the same time point,

^c^ P < 0.05.

### 3.3 Secondary outcome measures

#### 3.3.1 Comparison of inflammatory markers at different time points

**Figure 2:**
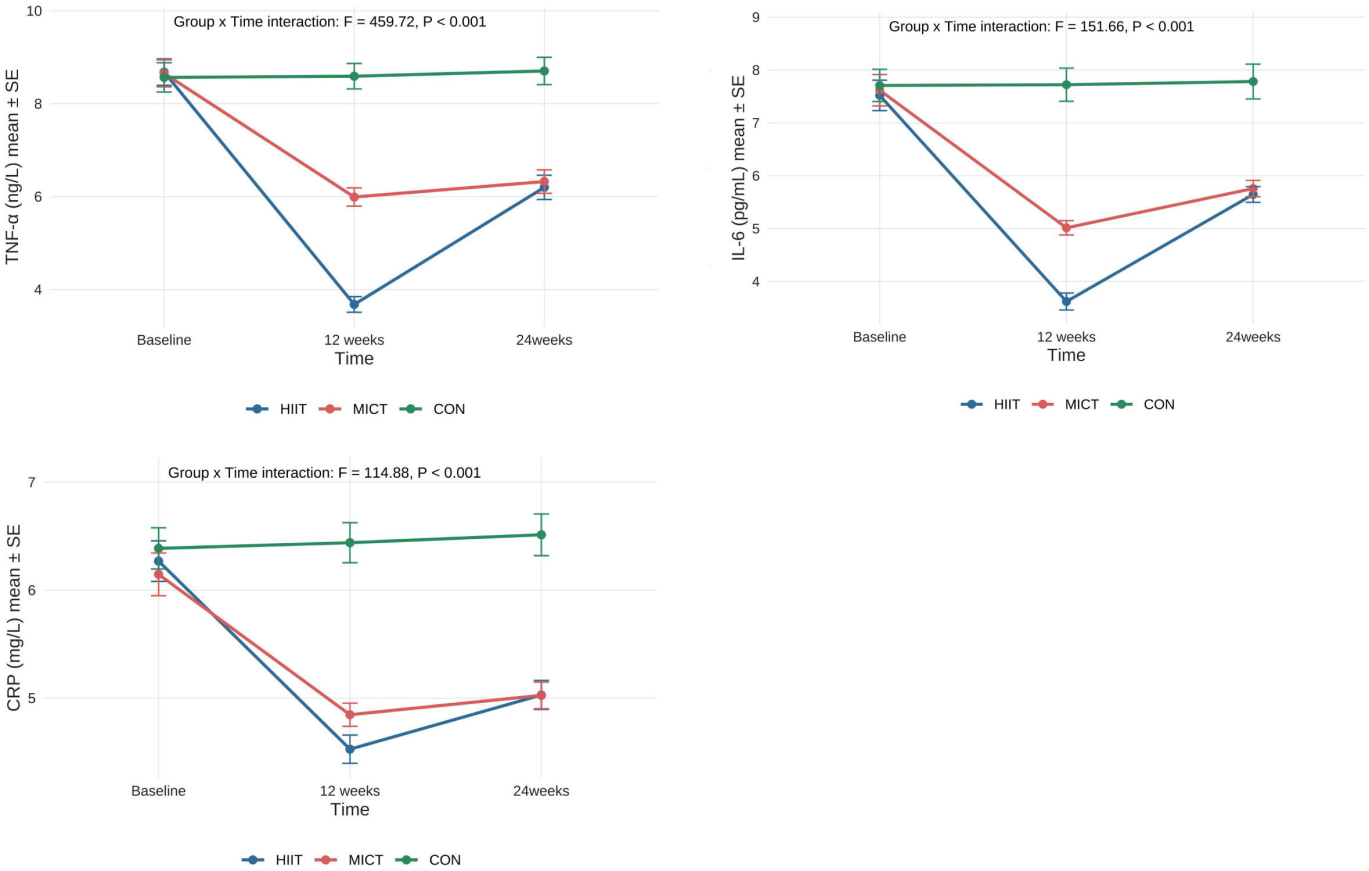
Trend chart showing the mean values of inflammatory markers at various time points.

The results of repeated measures analysis of variance indicated significant interaction effects between the time factor and the intervention group for TNF-α, IL-6, and CRP (all P < 0.001) (**Table 3**; **Figure 2**), suggesting that the effects of the various interventions on inflammatory markers exhibited different trends over time. Further analysis using Bonferroni *post-hoc* tests revealed that, in terms of the time effect, compared with pre-intervention levels, the HIIT and MICT groups showed significant reductions in TNF-α, IL-6, and CRP at the 12-week and 24-week follow-up visits (all P < 0.05), whereas no significant changes in these markers were observed in the control group at any time point (all P > 0.05). Analysis of between-group effects showed that, compared with the control group, both the HIIT and MICT groups exhibited significant reductions in TNF-α, IL-6, and CRP at the 12-week and 24-week follow-ups (all P < 0.05); compared with the MICT group, the HIIT group showed significant reductions in both TNF-α and IL-6 at the 12-week follow-up (both P < 0.05).

**Table 3:** Comparison of inflammatory markers among HIIT, MICT, and CON.

Variables	Baseline	12 weeks	24 weeks	Time×group F (P)	Partial η²	95% CI
TNF-α (ng/L)				459.716 (<0.001)	0.917	0.898-1.000
HIIT	8.68 ± 1.54	3.68 ± 0.91abc	6.20 ± 1.40ab			
MICT	8.65 ± 1.57	5.99 ± 1.06ab	6.33 ± 1.36ab			
CON	8.57 ± 1.67	8.59 ± 1.45	8.71 ± 1.56			
IL-6 (pg/mL)				151.664 (<0.001)	0.785	0.725-1.000
HIIT	7.52 ± 1.55	3.62 ± 0.86abc	5.65 ± 0.80ab			
MICT	7.62 ± 1.60	5.01 ± 0.73ab	5.76 ± 0.83ab			
CON	7.71 ± 1.61	7.72 ± 1.66	7.78 ± 1.75			
CRP (mg/L)				114.884 (<0.001)	0.735	0.663-1.000
HIIT	6.27 ± 1.01	4.53 ± 0.70ab	5.03 ± 0.72ab			
MICT	6.15 ± 1.07	4.85 ± 0.58ab	5.03 ± 0.66ab			
CON	6.39 ± 1.01	6.44 ± 0.98	6.51 ± 1.02			

Compared with pre-intervention levels within the group,

^a^ P < 0.05; compared with the control group at the same time point,

^b^ P < 0.05; compared with the MICT group at the same time point,

^c^ P < 0.05.

#### 3.3.2 Comparison of nerve conduction velocity, TCSS scores and Berg scores at various time points

**Figure 3:**
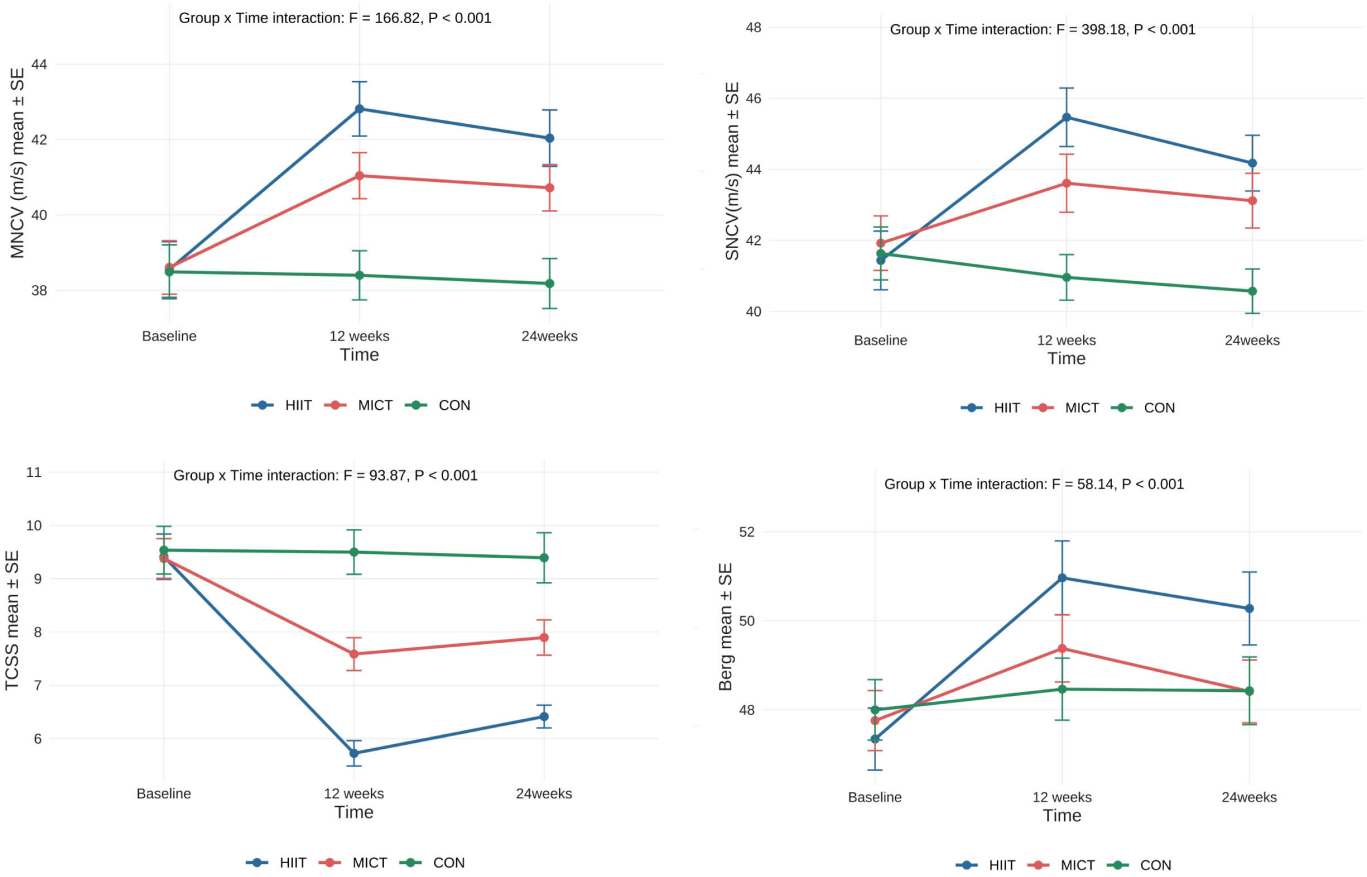
Trend graphs showing the mean values of nerve conduction velocity, TCSS scores and Berg scores at various time points.

The results of repeated measures analysis of variance indicated significant interaction effects between the time factor and intervention group for MNCV, SNCV, TCSS scores, and Berg scores (all P < 0.001) (**Table 4**; **Figure 3**), suggesting that the effects of the various interventions on MNCV, SNCV, TCSS scores, and Berg scores exhibited different trends over time. Further analysis using Bonferroni *post-hoc* tests revealed that compared with pre-intervention levels, both the HIIT and MICT groups showed significant improvements in MNCV and SNCV at the 12-week and 24-week follow-ups (all P < 0.05), whereas TCSS scores decreased significantly (all P < 0.05). The Berg score in the HIIT group increased significantly at all time points compared to pre-intervention levels (all P < 0.05), whereas no significant changes were observed in the control group for the aforementioned indicators at any time point (all P > 0.05). Analysis of between-group effects showed that compared with the control group, both the HIIT and MICT groups exhibited significant improvements in MNCV and SNCV at the 12-week and 24-week follow-ups (all P < 0.05) and significant reductions in TCSS scores (all P < 0.05). The Berg score in the HIIT group was significantly higher than that in the control group at all time points (all P < 0.05). Compared with the MICT group, the TCSS score in the HIIT group decreased significantly at both the 12-week and 24-week follow-ups (all P < 0.05).

**Table 4:** Comparison of nerve conduction velocity, TCSS score, and Berg score among HIIT, MICT, and CON.

Variables	Baseline	12 weeks	24 weeks	Time×group F (P)	Partial η²	95% CI
MNCV (m/s)				166.816 (<0.001)	0.801	0.752-1.000
HIIT	38.55 ± 3.98	42.82 ± 3.88^ab^	42.04 ± 4.03^ab^			
MICT	38.61 ± 3.82	41.04 ± 3.29^ab^	40.72 ± 3.31^ab^			
CON	38.49 ± 3.79	38.40 ± 3.45	38.18 ± 3.51			
SNCV (m/s)				398.179 (<0.001)	0.906	0.882-1.000
HIIT	41.43 ± 4.45	45.47 ± 4.43^ab^	44.18 ± 4.23^ab^			
MICT	41.92 ± 4.13	43.61 ± 4.40^ab^	43.12 ± 4.15^ab^			
CON	41.63 ± 3.95	40.96 ± 3.39	40.57 ± 3.30			
TCSS score				93.873 (<0.001)	0.694	0.623-1.000
HIIT	9.41 ± 2.29	5.72 ± 1.28^abc^	6.41 ± 1.15^abc^			
MICT	9.38 ± 2.01	7.59 ± 1.66^ab^	7.90 ± 1.78^ab^			
CON	9.54 ± 2.36	9.50 ± 2.20	9.39 ± 2.48			
Berg score				58.139 (<0.001)	0.584	0.497-1.000
HIIT	47.34 ± 3.75	50.97 ± 4.47^ab^	50.28 ± 4.42^ab^			
MICT	47.86 ± 3.63	49.18 ± 4.05	48.41 ± 3.79			
CON	48.00 ± 3.59	48.26 ± 3.69	48.43 ± 4.02			

Compared with pre-intervention levels within the group,

^a^ P < 0.05; compared with the control group at the same time point,

^b^ P < 0.05; compared with the MICT group at the same time point,

^c^ P < 0.05.

### 3.4 Adverse events and safety

Adverse events during the intervention and follow-up periods were recorded and compared among the three groups (**Table 5**). No severe exercise-related adverse events occurred during the study.

**Table 5:** Adverse events during the intervention and follow-up periods.

Adverse events	HIIT (n=29)	MICT(n=29)	CON(n=28)
Mild hypoglycemia episodes, n (%)	2 (6.9)	1 (3.4)	0
Foot-related problems, n (%)	0	0	0
Musculoskeletal pain/discomfort, n (%)	2 (6.9)	1 (3.4)	0
Cardiovascular symptoms during exercise, n (%)	1 (3.4)	1 (3.4)	0
Severe adverse events, n (%)	0	0	0
Withdrawal due to exercise intolerance, n (%)	0	0	0

Mild hypoglycemic episodes were observed in a small number of participants in both exercise groups and were relieved after carbohydrate supplementation without requiring medical intervention. No diabetic foot ulceration or severe cardiovascular events occurred. A few participants reported transient musculoskeletal discomfort during the early intervention period, which resolved after exercise adjustment. No participants withdrew from the study because of exercise intolerance or serious adverse events. Overall, both HIIT and MICT were well tolerated under supervised conditions with routine glucose and heart rate monitoring.

## 4 Discussion

This study, based on a repeated-measure design, systematically compared the dynamic effects of HIIT and MICT on indicators related to glucose metabolism, inflammatory responses, and neurological function in patients with DPN. The results showed that both HIIT and MICT were able, to a certain extent, to improve HbA1c, FPG and HOMA-IR levels, reduce the expression of inflammatory factors such as TNF-α, IL-6 and CRP, and promote the recovery of nerve conduction velocity and clinical functional improvement. Compared with MICT, HIIT demonstrated more significant and sustained advantages in certain key indicators, particularly in terms of HbA1c, FPG, TNF-α, IL-6 and TCSS scores. These findings suggest that different exercise intensity patterns exert differential regulatory effects on metabolic homeostasis and neurofunctional remodelling in patients with DPN, and that HIIT may exert a more significant intervention effect on the pathological progression of DPN through stronger metabolic stimulation and anti-inflammatory effects.

### 4.1 Impact of HIIT on glucose metabolism in patients with DPN

Glycemic control is the cornerstone of DPN health management; for every 1% reduction in HbA1c, the risk of diabetic microvascular complications decreases by 37% (18). The significant improvements in HbA1c and FPG observed in this study suggest that HIIT may have potential clinical value in slowing the progression of DPN and reducing the risk of microvascular complications. For patients with DPN, more effective glycaemic control may also further reduce clinical symptoms such as neuroischaemia, pain and sensory abnormalities, thereby improving overall disease management.The mechanism by which HIIT improves glucose metabolism may involve synergistic interactions among multiple organs. HIIT induces a significant excess post-exercise oxygen consumption (EPOC) effect, whereby corticosteroid levels remain elevated for a considerable period after exercise, and the body’s metabolic rate is maintained at a high level, thereby sustaining energy and glucose consumption (19). Skeletal muscle is the primary target organ for the body’s adaptive response to exercise stimuli and is also a key tissue for glucose uptake and metabolic regulation. HIIT, may by repeatedly activating AMP-activated protein kinase (AMPK) and silencing information regulator 1 (SIRT1), upregulates peroxisome proliferator-activated receptor gamma co-activator 1 alpha (PGC-1α), thereby strongly inducing the expression of mitochondrial oxidative phosphorylation-related enzyme systems, enhancing the oxidative capacity of muscle cells for fatty acids and glucose, and significantly improving insulin resistance (20). At the vascular level, the intermittent shear stress and metabolic demands may induced by HIIT upregulate the expression and activity of vascular endothelial growth factor (VEGF) and endothelial nitric oxide synthase (eNOS), driving angiogenesis and increasing microvascular perfusion in skeletal muscle, whilst simultaneously promoting the bioavailability of nitric oxide (NO) and restoring endothelial function impaired by oxidative stress, thereby ensuring the transendothelial transport of insulin and glucose to muscle cells (21). In adipose tissue, HIIT may inhibit NADPH oxidase activation and enhances the endogenous antioxidant system, thereby alleviating oxidative stress. Concurrently, it downregulates pro-inflammatory factors such as monocyte chemotactic protein-1 (MCP-1), upregulates adiponectin expression, reprograms lipid metabolic pathways, reduces circulating free fatty acid levels, and corrects ectopic lipid accumulation (22). The aforementioned improvements in mitochondrial biogenesis, microvascular remodelling, and oxidative stress in adipose tissue are interlinked, collectively constituting the multi-tissue molecular basis by which HIIT enhances systemic insulin sensitivity and optimizes glucose metabolic homeostasis. This study found that, following a 12-week intervention, HIIT demonstrated a more pronounced advantage over MICT in improving HbA1c and FPG, suggesting that higher exercise intensity may confer additional benefits in promoting glucose metabolic regulation. These findings suggest that, provided safety monitoring and personalised exercise programmes are in place, HIIT holds promise as a more effective exercise strategy for optimising metabolic control in patients with DPN, particularly for those who require metabolic improvement within a short timeframe. However, in a study by Findikoglu G et al. (23) employing exercise protocols with equal energy expenditure, no significant differences were observed between HIIT and MICT in terms of HbA1c, FPG, insulin levels, or insulin sensitivity. The heterogeneity in these findings may be attributed to factors such as the control of total exercise load, participants’ baseline metabolic status, the duration of the intervention, and exercise adherence. This also suggests that the metabolic advantages of HIIT may depend not only on exercise intensity itself, but also on actual energy expenditure and individual metabolic adaptability.

### 4.2 Impact of HIIT on inflammatory responses in patients with DPN

This study found that following HIIT intervention, serum levels of tumor necrosis factor-α (TNF-α), interleukin-6 (IL-6), and C-reactive protein (CRP) in patients all decreased significantly, suggesting that HIIT can effectively alleviate the chronic low-grade inflammatory state in patients with DPN; this result is largely consistent with the findings reported by Sabouri et al. (24). As chronic inflammation is closely associated with pain, sensory abnormalities and worsening neurological function in patients with DPN, a reduction in inflammatory markers may indicate an improvement in the patients’ neural microenvironment and may further reduce the risk of disease progression. Persistent chronic inflammatory responses are key pathological mechanisms underlying the onset and progression of DPN; chronic low-grade inflammation and persistent hyperglycemia promote endothelial dysfunction, exacerbate ischemia and hypoxia in the nerve endothelium, and disrupt normal neuronal function (25). HIIT may modulate inflammation associated with diabetic peripheral neuropathy through multilevel molecular mechanisms, primarily by inhibiting the proinflammatory cascade mediated by the nuclear factor E type kB (NF-κB) signalling pathway, activating the AMP-activated protein kinase (AMPK) signalling pathway to improve metabolic homeostasis, and reducing oxidative stress levels by promoting mitochondrial functional remodelling (26). HIIT is more effective at inducing skeletal muscle, acting as an endocrine organ, to release a series of anti-inflammatory cytokines potentially, such as interleukin-1 receptor antagonist (IL-1ra) and interleukin-10 (IL-10), thereby counteracting the effects of proinflammatory factors (27). Furthermore, HIIT exerts anti-inflammatory effects at both systemic and local microenvironmental levels by modulating the secretion profile of adipokines, improving vascular endothelial function, and inhibiting the activation of local immune cells in the nervous system, thereby potentially exerting a protective effect against the progression of nerve damage (28). This study found that, compared with MICT, HIIT may exhibit more pronounced anti-inflammatory effects in the short term; during a 12-week short-term intervention, HIIT demonstrated a more pronounced inhibitory effect on proinflammatory factors, such as TNF-α and IL-6, whereas after a 24-week follow-up, the differences between the two exercise modalities in terms of inflammatory improvement gradually diminished, suggesting that its anti-inflammatory effects are time-dependent, which may be related to its ability to more strongly activate energy metabolism-related signalling pathways and induce more pronounced adaptive responses to oxidative stress (29).

### 4.3 Impact of HIIT on on nerve conduction velocity, TCSS score and Berg score in patients with DPN

This study observed that following the HIIT intervention, both the MNCV and SNCV of the common peroneal nerve improved in the patients. Concurrently, in terms of the TCSS and Berg scales, high-intensity interval training demonstrated more pronounced short-term improvements. Notably, these functional improvements are highly clinically relevant. The increase in nerve conduction velocity and the reduction in TCSS scores suggest that the extent of nerve damage in patients may have improved, while the enhancement in balance may help to reduce the risk of falls, improve gait stability, and enhance the ability to perform activities of daily living, thereby improving patients’ quality of life and functional independence. Previous studies have demonstrated that diabetes-related neuromuscular dysfunction involves impaired motor unit activation, reduced muscle quality, altered proprioceptive feedback, and deficits in postural control, all of which contribute to mobility limitations and increased fall risk in patients with DPN. Therefore, the improvements in nerve conduction velocity, TCSS scores, and Berg Balance Scale scores observed in the present study may reflect not only metabolic improvement but also enhanced neuromuscular coordination and motor control adaptation induced by repeated high-intensity exercise stimuli (30). This indicates that HIIT not only improved physiological indicators but also translated into tangible functional benefits. The improvements in nerve conduction velocity and functional performance may primarily involve the coordinated regulation of neural structural protection and functional reconstruction. As an external stimulus, HIIT may upregulate the expression of key proteins that promote neurogenesis and synaptic plasticity, such as brain-derived neurotrophic factor (BDNF), thereby supporting axonal regeneration and myelin repair. It also activates energy metabolism-related signalling pathways (such as the AMPK pathway) to improve mitochondrial function and ATP production efficiency and reduces inflammatory responses and oxidative stress damage by inhibiting the NF-κB signalling pathway (31, 32). HIIT may alleviate neural ischemia by improving vascular endothelial function and neural microcirculatory perfusion, thereby increasing neural blood flow and nutrient supply (33), which may precede substantial structural nerve regeneration. Therefore, short-term improvements in electrophysiological parameters may reflect functional adaptations rather than complete neural repair. However, Bönhof et al. (34) reported that 12 weeks of supervised HIIT improved cardiovascular autonomic function in individuals with type 2 diabetes but did not significantly improve nerve conduction studies, quantitative sensory testing, or intraepidermal nerve fibre density. Several factors may explain these differences. First, our study specifically enrolled patients with clinically diagnosed DPN, whereas the population investigated by Bönhof et al. included individuals in overweight men with type 2 diabetes who may have exhibited different degrees of neuropathic involvement. Consequently, participants in the present study may have had greater potential for functional improvement. Second, the outcome measures differed between studies. While intraepidermal nerve fibre density primarily reflects structural nerve regeneration, nerve conduction velocity may be more sensitive to early functional adaptations related to improved neural blood flow, metabolic control, and axonal function. Therefore, improvements in electrophysiological function may precede detectable structural changes. Finally, differences in baseline characteristics, intervention implementation, and statistical power may also have contributed to the observed discrepancies.Taken together, the available evidence suggests that HIIT may exert beneficial effects on neural function; however, the extent to which these improvements translate into structural nerve regeneration remains uncertain and warrants further investigation.

In addition, repeated high-intensity muscular contractions may enhance afferent sensory input and motor output synchronization, contributing to improved postural control, gait stability, and dynamic balance function. Exercise-induced improvements in peripheral circulation, axonal excitability, and neural metabolic support may further facilitate recovery of nerve conduction function. These mechanisms may partially explain the superior short-term improvements in TCSS and balance outcomes observed in the HIIT group compared with the MICT group (35).

## 5 Limitations

Although this study yielded positive results, several limitations remain. The sample size was relatively small; larger-scale, multicenter studies are required in the future to further validate the generalizability of these findings. The intervention period was 12 weeks; whether longer-term HIIT interventions would yield additional benefits, or whether a plateau exists, requires clarification through long-term follow-up studies. The control group received only health education and telephone follow-up rather than an attention-matched supervised intervention. Therefore, differences in participant contact time, supervision intensity, behavioral reinforcement, and exercise engagement between groups may have partially contributed to the observed effects. Future studies should incorporate attention-matched control conditions or sham exercise interventions to better distinguish the specific physiological benefits of HIIT from nonspecific supervision or behavioral effects.This study primarily focused on blood biochemical and functional parameters, although several mechanistic pathways discussed above, including BDNF signaling, AMPK activation, mitochondrial remodeling, VEGF/eNOS-mediated vascular adaptations, and neuroregenerative processes, were not directly measured in the present study. Therefore, these mechanisms should be considered hypothetical explanations based on previous experimental and clinical evidence rather than direct conclusions derived from our data. Future studies incorporating molecular, neurophysiological, and imaging biomarkers are warranted to further elucidate the biological mechanisms underlying the observed benefits of HIIT in patients with DPN. Finally, for safety reasons, participants were rigorously screened and those with severe cardiovascular complications were excluded; consequently, the study’s conclusions may not apply to all DPN patients, and individualized exercise prescriptions and enhanced monitoring should be implemented when applying these findings in clinical practice.

## 6 Conclusions

Both HIIT and MICT were associated with improvements in metabolic control, inflammatory markers, and selected neuropathy outcomes in patients with DPN. HIIT showed greater benefit for some measures, but larger adequately powered trials with rigorous statistical handling and safety reporting are needed before concluding overall superiority.

## Data Availability

The original contributions presented in the study are included in the article/supplementary material. Further inquiries can be directed to the corresponding author.
